# Effects of Upper Blepharoplasty Techniques on Headaches, Eyebrow Position, and Electromyographic Outcomes: A Randomized Controlled Trial

**DOI:** 10.3390/ijerph20021559

**Published:** 2023-01-14

**Authors:** Maria H.J. Hollander, Johannes H. van der Hoeven, Koen H.M. Verdonschot, Konstantina Delli, Arjan Vissink, Johan Jansma, Rutger H. Schepers

**Affiliations:** 1 Department of Oral and Maxillofacial Surgery, University of Groningen and University Medical Center Groningen (UMCG), 9700 RB Groningen, The Netherlands; 2 Department of Clinical Neurophysiology, University of Groningen and University Medical Center Groningen (UMCG), 9700 RB Groningen, The Netherlands; 3 Faculty of Science and Technology, Department of Technical Medicine, University of Twente, 7522 NB Enschede, The Netherlands

**Keywords:** blepharoplasty, eyelid correction, EMG, electromyography, headache

## Abstract

The aim of this study was to assess changes in headaches, eyebrow height, and electromyographic (EMG) outcomes of the frontalis and orbicularis oculi muscles, after an upper blepharoplasty with or without resecting a strip of orbicularis oculi muscle. In a randomized controlled trial, 54 patients received an upper blepharoplasty involving either only removing skin (group A) or removing skin with an additional strip of orbicularis muscle (group B). Preoperative, and 6 and 12 months postoperative headache complaints were assessed using the HIT-6 scores and eyebrow heights were measured on standardised photographs. Surface EMG measurements, i.e., electrical activity and muscle fatigue, were assessed for the frontalis and orbicularis oculi muscles preoperatively and 2, 6, and 12 months postoperatively. Significantly fewer headaches were reported following a blepharoplasty. The eyebrow height had decreased, but did not differ between groups. Regarding the surface EMG measurements, only group A’s frontalis muscle electrical activity had decreased significantly during maximal contraction 12 months after surgery (80 vs. 39 mV, *p* = 0.026). Fatigue of both the frontalis and the orbicularis oculi muscles did not change significantly postoperatively compared to baseline. EMG differences between groups were minor and clinically insignificant. The eyebrow height decreased and patients reported less headaches after upper blepharoplasty irrespective of the used technique.

## 1. Introduction

Patients with dermatochalasis of the upper eyelids often elevate their eyebrows by recruiting the frontalis muscles in order to compensate for the visual field obstruction caused by sagging of the upper eyelid skin. This elevation may be associated with an increase in frontalis muscle electrical activity [1] and might cause other problems such as tension-type headaches due to constant muscle activation or insufficient relaxation [2]. This relationship is controversial [3].

An upper blepharoplasty can be a solution for dermatochalasis of the eyelids, providing general improvements in functional complaints [4] and an enhancement in facial beauty [5]. During treatment, the redundant skin is removed thereby alleviating any possible visual field obstructions. Postoperatively, it is no longer necessary to elevate and activate the eyebrow muscles. Theoretically, feedback from the brain to the frontalis muscles to continue to elevate the eyebrow is lost. This may result in lowering of the brows and softening of the forehead wrinkles. In addition, this relaxation of the frontalis muscle might be the reason for the clinical observation that some patients experience significantly fewer tension-type headaches after an upper blepharoplasty.

The literature is inconsistent regarding what happens to the eyebrow height after an upper blepharoplasty [4]. Although they tend to move down, the extent and the influence on aesthetic and functional outcomes are unknown. The lowering of the eyebrows is theoretically regarded as the result of either the diminished need to elevate the forehead as a compensatory mechanism for the elevation of the eyebrows, and thereby the upper eyelids [6], or the mechanical depression of the eyebrow by removing a large amount of eyelid tissue or by more invasive surgery. In theory, when excising more tissue, such as with the traditional upper blepharoplasty technique, more scarring might occur which, in turn, might lead to pulling the eyebrows down. As to whether the lowering of the eyebrows can be explained by changes in muscle activity, further research is needed. In addition, it is unclear whether orbicularis oculi function is compromised after excising a strip of it during an upper blepharoplasty. Traditionally, an upper blepharoplasty entailed the removal of redundant skin with the underlying orbicularis oculi muscle and/or protruding fat. Modern surgical insights emphasize volume preservation and sparing of the orbicularis oculi muscle [7]. Therefore, the more conservative surgical upper blepharoplasty, which consists of only removing redundant skin, is gaining popularity.

The question arises whether a cosmetic upper blepharoplasty has an effect on the eyebrow position, frontalis muscle activation/fatigue, and possibly headaches experienced by patients, and whether there is a relationship between these variables. Thus, the aim of this RCT was to assess the electrical activity of the upper facial muscles, eyebrow position, and tension-type headaches after two surgical upper blepharoplasty techniques.

## 2. Materials and Methods

### 2.1. Study Design

A prospective single-centre randomized, double-blind, controlled trial investigated eyebrow position, electrical activity of the upper facial muscles, and headaches after upper blepharoplasties. The study protocol was approved by the institutional review board (METc2017/451), following the Declaration of Helsinki Ethical Principles for Medical Research Involving Human Subjects, and was registered in the Netherlands Trial Register (ID NL7886). Written informed consent was obtained from all the study participants.

### 2.2. Study Population

**Figure 1:**
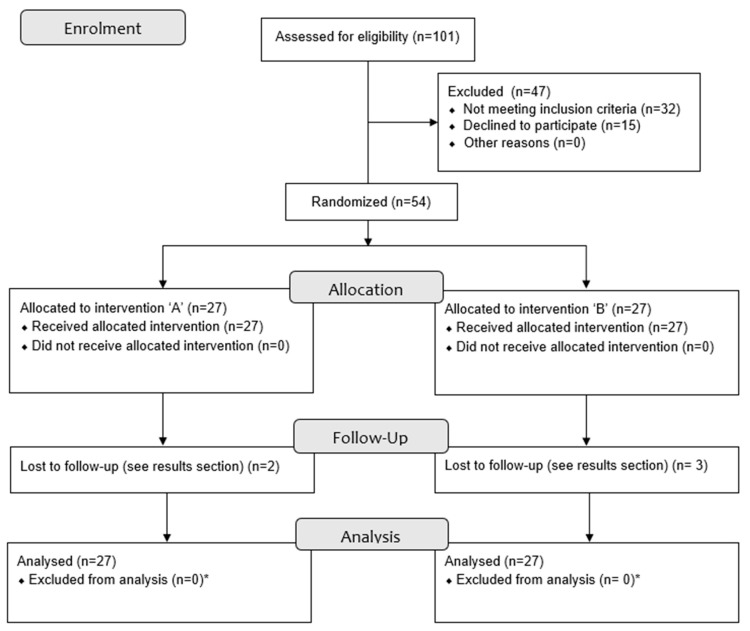
Flow diagram of participant enrolment. * Only the ‘lost to follow up’ missing values were excluded from analysis.

All consecutive Caucasian patients between 30 and 70 years of age who consulted the Department of Oral and Maxillofacial Surgery at the University Medical Center Groningen for an upper blepharoplasty, between February 2018 and October 2019, were asked to participate (Figure 1). Patients were included if they showed dermatochalasis of both upper eyelids and an upper eyelid blepharoplasty was indicated. The consultations were performed by two maxillofacial surgeons (J.J., R.H.S.) with extensive experience in upper blepharoplasties. The patients had to be fluent in Dutch in order to fully understand the Dutch questionnaires. Patients were excluded if they had a history of ocular or orbital trauma, trauma of the upper half of the face, a history of eyelid- or eyebrow-region surgery, had been subjected to other cosmetic surgical or non-surgical procedures, had ophthalmic disease, or suffered from blepharoptosis. Patients suffering from any other medical condition that could affect the electromyogram were also excluded.

### 2.3. Blinding and Randomization

The eligible participants were randomly assigned to treatment group ‘A’ (resection of skin only) or ‘B’ (resection of skin and a strip of underlying orbicularis oculi muscle) according to the list created prior to the start of the study by a randomization computer tool (Sealed Envelope Ltd., 2017, London, UK). The participants received a unique code in consecutive order, i.e., the first included participant received the first code on the list. The investigators and participants were blinded in that the latter were informed about both surgical procedures, but did not know which treatment they had undergone, and received identical information about the possible postoperative course of events. Only the surgeons knew which was treatment ‘A’ or ‘B’ until the completion of the trial.

### 2.4. Outcomes

Demographic data were recorded including age, gender, medical history, and use of medication. The severity of the dermatochalasis was assessed before the upper blepharoplasty and categorized according to a 4-level photonumerical severity scale using anatomical cut-off points: normal, if the upper eyelid skin was not touching the eyelashes; mild, if the upper eyelid skin was touching the eyelashes; moderate, if the upper eyelid skin was hanging over the eyelashes; and severe, if the upper eyelid skin was hanging over the eye [8]. The removed tissue was weighed per eye and recorded in grams.

#### 2.4.1. Headache Impact

The Headache Impact Test 6 (HIT 6), a questionnaire consisting of 6 items (pain intensity, social functioning, role functioning, vitality, cognitive functioning, and psychological distress), was used to assess headaches [9,10,11]. Each question can have a score between 6 and 13, so the minimum score is 36 and the maximum score is 78. A score of 60 or more is indicative of extremely severe headaches, a score between 56–59 severe headaches, a score between 50–55 moderate headaches, and a score between 36 and 49 indicates no to mild headaches [12]. The questionnaire was completed directly preoperatively and postoperatively at 6 and 12 months.

#### 2.4.2. Eyebrow Height

**Figure 2:**
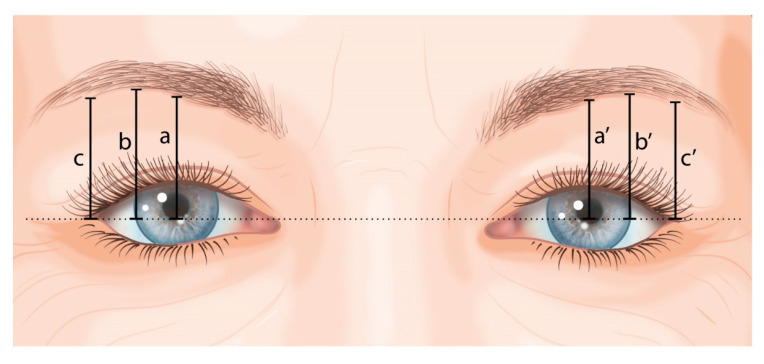
Eyebrow height measurements.

Standardised digital 2D photographs of the primary gaze were taken just before the surgery and 6 and 12 months postoperatively, with the head in a natural position, to assess eyebrow height. Each photograph was taken by the same researcher (M.H.J.H.) under the same lighting conditions, at a fixed distance and with the same camera (Nikon D5600 AF-S DX NIKKOR VR, Minato, Tokyo, Japan). To account for size discrepancy between photographs, a horizontal visible iris diameter of 11.71 mm was used for calibration purposes [13,14,15,16]. The distances on the photographs were measured digitally using the NIH ImageJ software (Version 1.53a, National Institutes of Health, Bethesda, MD, USA) as illustrated in Figure 2. First, a horizontal line was drawn through the exocanthion. Then, the following distances were measured:

- a and a’: vertical line to the lower boundary of the eyebrow at the pupil’s midline;
- b and b’: vertical line to the lower boundary of the eyebrow at the lateral border of the iris;
- c and c’: vertical line to the lower boundary of the eyebrow at the exocanthion.

The eyebrow height measurements were performed by one researcher (M.H.J.H.) and then repeated by an independent researcher (M.C.) to assess inter-observer variability.

#### 2.4.3. Electromyography

**Figure 3:**
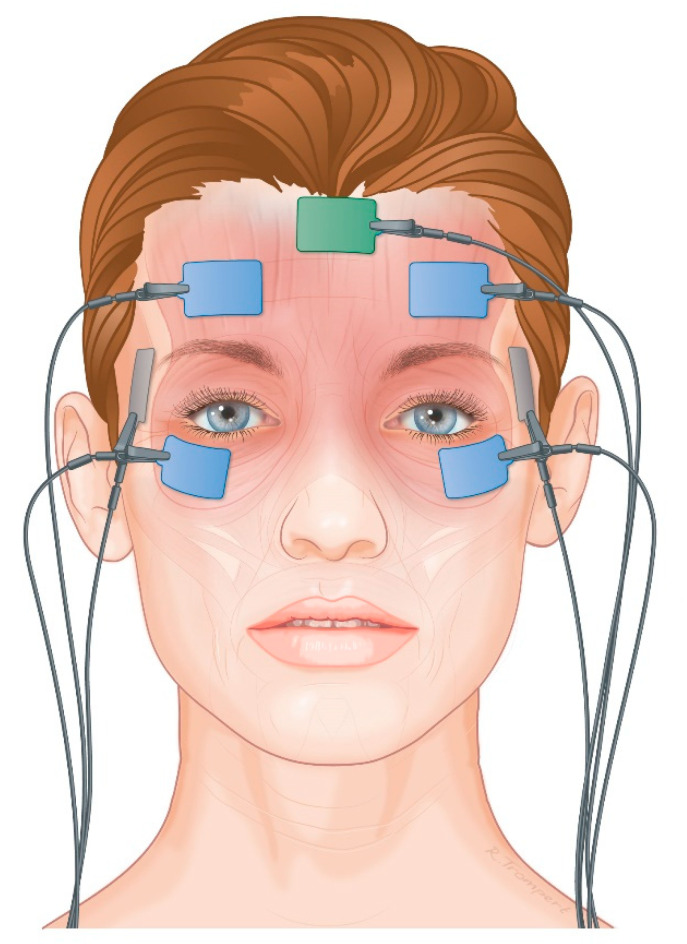
Surface electromyography electrode placement.

**Figure 4:**
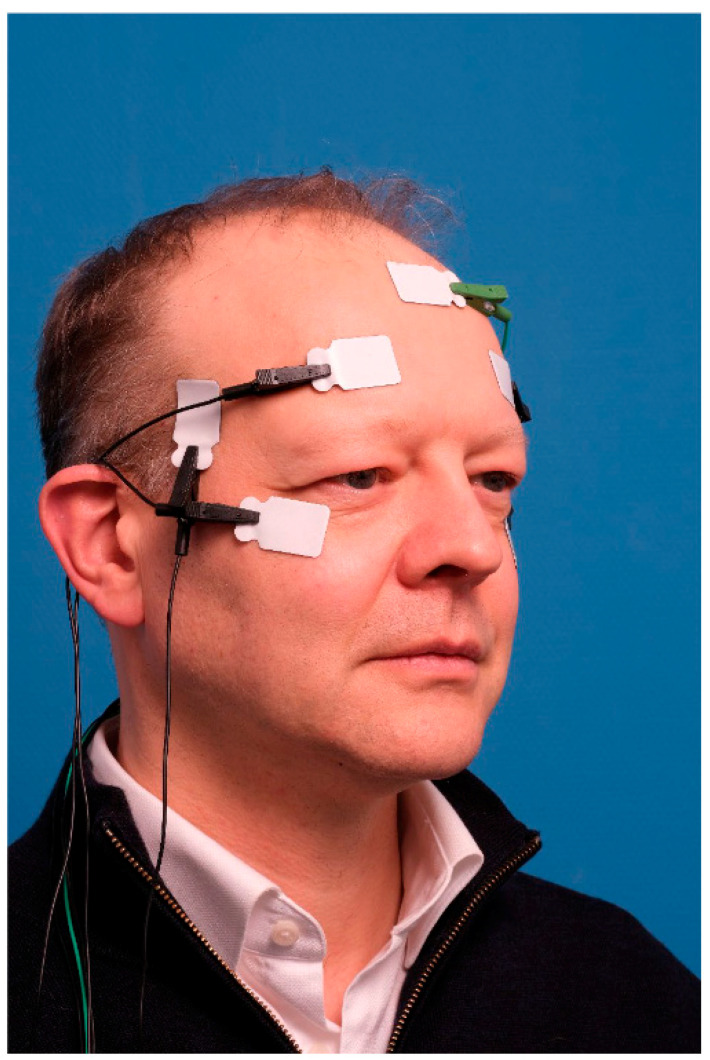
Photograph of EMG placement.

Directly before the blepharoplasty and 2, 6, and 12 months postoperatively, an electromyography of the frontalis muscles and orbicularis oculi muscles was performed. All the electromyography signals were recorded (unfiltered) with the BrainRT system with a 1 kHz reach, and a Duo 44 US EEG-PSG amplifier (Natus Europe GmbH, Planegg, Germany), without a notch filter. First, the skin of the upper face was cleaned with alcohol and abrasive gauze before attaching the surface electrodes. The Ag/AgCl-electrodes were rectangular (22 × 32 mm; 3 M Red Dot™ 3 M Center, St. Paul Minnesota, MI, USA) and contained conductive adhesive. The reference electrodes were attached to the skin covering the temporalis muscles (both sides; Figure 3 and Figure 4). The active electrodes were attached to the muscle belly of the frontalis muscles right above the pupils and 15 mm above the eyebrows on both sides. The grounding was attached midline, just below the hairline. In addition, in order to measure the electrical activity of the orbicularis oculi muscle, another surface electrode was attached to the laterocaudal part of the orbicularis oculi (left and right eye). The patients sat in an upright position and were instructed to look at a fixed point on the wall. Then, the different tasks were rehearsed, consisting of closing the eyes gently, raising eyebrows maximally, with a neutral gaze (looking at the fixed point), and closing the eyes firmly. This was repeated and the electromyography signal was stored together with the integrated video footage of the face during the tasks in the BrainRT software (RT Software Suite version 3.1, O.S.G. bvba, Kontich, Belgium). We recorded 10 s of every task, of which 5 s were used for analysis [17]. The first 2 s were excluded from the analysis due to movement artefacts when performing the tasks, as well as the last 3 s. During the analysis, the video footage was checked in order to confirm correct movement execution.

In order to assess whether the muscle fatigue was caused by constantly raising the eyebrows preoperatively, multiple aspects of the EMGs were evaluated. During isometric contraction, muscle fatigue causes a decrease in the motor unit firing rate and the power density shift to lower frequencies. Then, additional fibres are recruited to maintain the muscle contraction which results in increased EMG amplitude and RMS (root-mean-square) values [18]. We therefore hypothesized that after blepharoplasty, electrical activity and muscle fatigue of the frontalis muscle might be less during the same isometric muscle contraction, since the constant raising of the eyebrow is no longer needed. Isometric contraction of the frontalis muscle was assessed by raising the eyebrow maximally, and the orbicularis oculi muscles by closing the eyes firmly. To assess these aspects, the root-mean-square (RMS) and the median frequency (Fmed) of the acquired EMG episodes were calculated and used to evaluate the electrical activity (RMS) and local fatigue (Fmed) of the muscles, which were processed by Matlab (version R2020b, The MathWorks, Inc., Natick, MA, USA). The EMG signal was analysed using the root-mean-square (RMS) method, which represents the square root of the average squared power of the EMG signal over a given period of time. To assess muscle fatigue, the median frequency was evaluated during the same 5 s of the surface EMGs (sEMGs). In addition, to assess the Fmed shift in more detail, the median frequency of the 3rd and 9th second (of the 10 recorded seconds during maximal contraction) were calculated and compared.

During the Matlab processing, filters were applied, i.e., a high-pass filter of 20 Hz as recommended by Van Boxtel et al. [19] and a low-pass filter frequency of 300 Hz. These filters were also chosen based on the visual interpretation of the EMG signals in BrainRT, which showed that all the signals were within these limits. In addition, a Butterworth filter of 50 Hz (and its harmonics) was used to compensate for the standard frequency of Europe’s electricity grid.

Then, a proportional index was provided by RMS/maximal amplitude of the maximal contraction to normalise the frontalis and orbicularis oculi activity values among the individuals.

### 2.5. Surgical Procedure

The upper blepharoplasties were performed by two surgeons (J.J., R.H.S.) in an outpatient environment. The surgical procedure was standardised prior to the study. The patients underwent the removal of upper eyelid skin only (group A) or the additional removal of a strip of orbicularis oculi muscle (group B); all the other steps were identical. The surgical landmarks and planned skin excisions were marked on the patient whilst in an upright position. Approximately 1.7 mL of Ultracaine DS Forte (40 mg Articain, 10 µg Epinephrine per mL), a local anaesthetic fluid, was injected subcutaneously per side. A scalpel was used to remove the marked excess upper eyelid skin and, in group B, 3–4 mm of the underlying orbicularis oculi muscle. The orbital septum was coagulated and the muscle edges were approximated with bipolar coagulation. The skin was sutured with Ethilon 6-0 (Ethicon, Cornelia, Georgia, GA, USA) intracutaneously in a running fashion and adhesive suture strips were placed. When indicated, the patients underwent removal of a significant amount of protruding medial fat.

### 2.6. Statistical Analysis

Twenty-seven patients were needed per treatment group to detect a difference of 8.3 in the HIT-6 score between groups A and B at 6 and 12 months, with a two-sided 5% significance level and a power of 85%, allowing for a 15% attrition rate and 10% for possible non-parametric testing (G* Power version 3.1.9.6, University of Kiel, Germany). The mean HIT-6 score is based on pre-and postoperative differences between two groups, i.e., the blepharoplasty and ptosis surgery groups [20].

The data were analysed using IBM SPSS Statistics version 26.0 (IBM Corp., Armonk, NY, USA). The Shapiro–Wilk test, Kolmogorov–Smirnov test, and graphical interpretation of Q–Q plots were used to determine the distribution of the data. All the tests were carried out for both sides of the patients’ faces (left and right) and were included in the data set. The independent samples t-test, Chi Square test, and Fisher’s exact test were applied where appropriate to test baseline differences between the groups.

Pre- and post-blepharoplasty differences in eyebrow height and HIT-6 score within groups were analysed using the Friedman test followed by pairwise comparisons, and Bonferroni adjusted *p*-values were applied. Differences between groups A and B regarding HIT-6 score, baseline HIT-6 scores, gender, age, dermatochalasis severity score, and removed tissue during surgery were evaluated using generalized estimating equations (GEE). All the residuals showed a Gaussian distribution and both models had a lowest information criterion in the exchangeable correlation structure. Additionally, the ‘responders’’ baseline HIT-6 scores were compared, i.e., the participants who displayed a decrease of ≥8 points on the HIT-6 score postoperatively [21], and then the ‘non-responders’’ scores, i.e., the participants who displayed a decrease of <8 points on the HIT-6 score postoperatively, using the Mann-Whitney U test.

Differences between groups A and B regarding eyebrow height in millimetres, baseline eyebrow height, gender, age, dermatochalasis severity score, and amount of removed tissue during surgery were also evaluated using GEE. The residuals showed a Gaussian distribution and the model with the lowest information criterion was used (i.e., m-dependent for the a and b landmarks, exchangeable correlation structure for landmark c).

The differences in eyebrow height change between the landmarks were also evaluated using the Friedman test with pairwise comparisons and by applying Bonferroni adjusted *p*-values.

To assess inter-observer agreement in measuring patients’ eyebrow height, all the measurements were performed by two raters (M.H.J.H. and M.C.) and intraclass correlation coefficient (ICC, two-way mixed effects model, single measurement, absolute agreement) was calculated. All the patients’ eyebrow height measurements were repeated to provide an intraclass correlation coefficient (ICC, two-way mixed effects model, single measurement, absolute agreement). The ICC values were interpreted as follows: 0.00–0.20, poor; 0.20–0.40, fair; 0.40–0.60, moderate; 0,60–0.80, good; 0.80–1.00, excellent [22].

The Friedman test was used to compare the post-surgical RMS, median frequencies, and the index (RMS/maximal amplitude) with the preoperative EMGs within each treatment group. Subsequently, a post-hoc test was carried out and Bonferroni adjusted *p*-values were applied.

The differences between groups A and B were evaluated using GEE. The GEE model included the EMG values, baseline sEMG values, gender, age, dermatochalasis severity score, and the amount of tissue removed during surgery. The residuals showed a Gaussian distribution, and the model with the lowest information criterion was used (i.e., exchangeable correlation structure). Only the RMS and median frequency values were transformed (log10) to achieve a Gaussian distribution of the residuals.

The correlation between the pre- and postoperative change in EMG values (mean frontalis muscle RMS of right and left eye), eyebrow height (mean height at the b and ‘b landmarks), and HIT-6 score was analysed with the Spearman correlation coefficient. The correlation coefficients, r values, were interpreted as follows: between 0–0.19, very weak; 0.2–0.39, weak; 0.40–0.59, moderate; 0.6–0.79, strong; and 0.8–1, very strong [23].

In addition, the baseline variables were correlated with changes in any other variable during the follow-up.

The baseline variables were EMG values (mean frontalis muscle RMS of right and left eye), eyebrow height (mean height at the b and ‘b landmarks), or HIT-6 score. The pre-and both the 6- and 12-month postoperative changes in these variables were used. This was done in order to investigate if baseline values could ‘predict’ the change in outcomes.

## 3. Results

**Figure 5:**
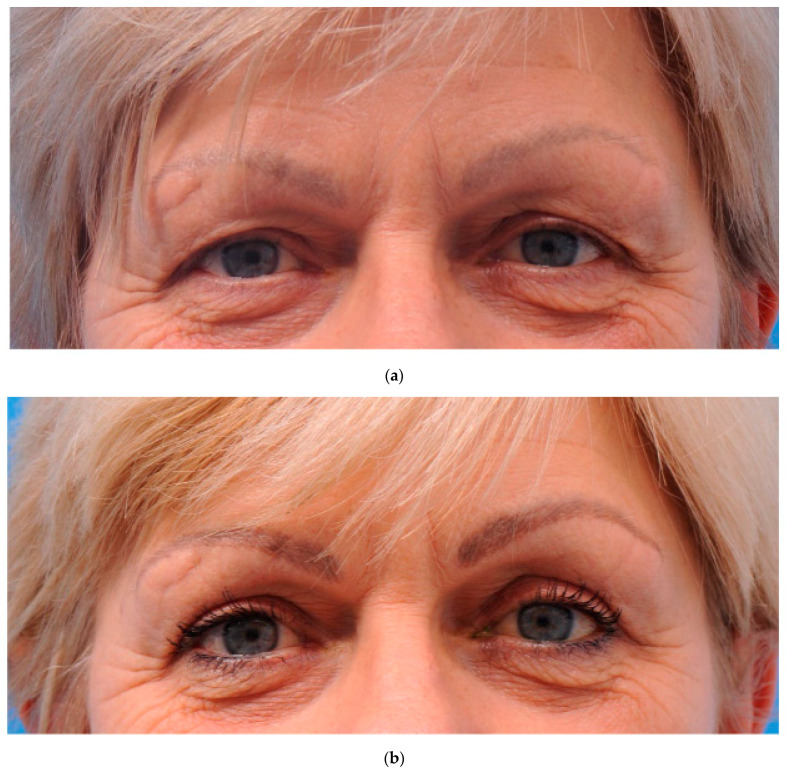
(**a**) Preoperative photograph of a participant from group A (skin only). (**b**) Photograph 12 months after upper eyelid blepharoplasty (group A; skin only).

**Figure 6:**
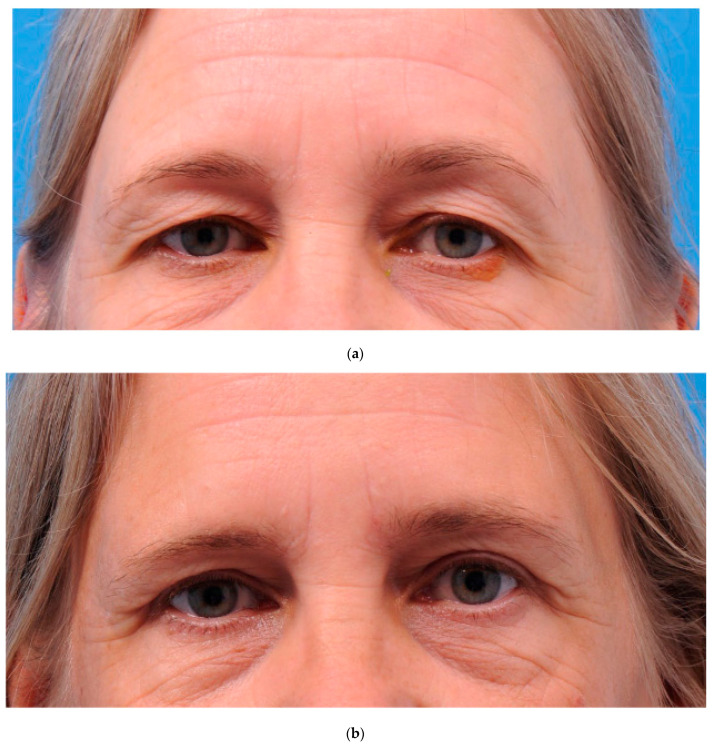
(**a**) Preoperative photograph of a participant from group B (skin/muscle). (**b**) Photograph 12 months after upper eyelid blepharoplasty (group B; skin/muscle).

Table 1 shows the characteristics of the 54 patients divided between groups A and B). The patients’ characteristics were comparable at baseline. A total of five female patients were excluded from the analysis: two patients (group B) were lost to the 2-month and 12-month follow-ups, two patients (group A) were excluded after the 6-month follow-up visit due to burn-out and multiple health problems related to a dysregulated diabetes mellitus, and one patient (group B) was excluded from the 12-month analysis because of her wish to correct the scarred tissue of one eyelid shortly after the initial procedure. The latter patient’s sutures had become loose which resulted in a widened scar that was corrected after the 6-month follow-up visit. The participants underwent upper blepharoplasty mainly for cosmetic reasons. For both procedures, a representative pre-and postoperative photograph are shown in Figure 5 and Figure 6.

### 3.1. HIT-6

The median HIT-6 scores are displayed in Table 2. There were no significant differences in HIT-6 scores between groups A and B during the 6- and 12-month follow-ups. Both groups demonstrated a significant improvement (group A *p* = 0.003; group B *p* = 0.029) in HIT-6 scores at the 12-month follow-up compared to baseline.

The responders (participants with a decrease of ≥8 points on the HIT-6 score postoperatively compared to baseline) showed a significantly higher baseline HIT-6 score (baseline median {Q1;Q3] HIT-6 score responder: 49 [46;61]; non-responder: 40 [37;43]) compared to the non-responders (*p* < 0.001).

### 3.2. Eyebrow Height

The inter-observer reliability of the eyebrow height measurements showed an excellent ICC of 0.967 (*p* < 0.001; 95% CI 0.917–0.986). Eyebrow heights were not significantly different at the 6- and 12-month follow-ups between groups A and B (Table 3). All the median postoperative eyebrow height measurements were significantly lower compared to baseline; the median eyebrow height decreased between 1.4 to 4.3 mm (Table 4). This applied to all the landmarks. When comparing the baseline and post-operatively measured landmarks, no significant differences were found in the change in eyebrow height (at the 6-month follow up, group A’s *p* = 0.936; group B’s *p* = 0.193; at the 12-month follow up, group A’s *p* = 0.938; group B’s *p* = 0.624).

### 3.3. Electromyography

Group A’s frontalis muscle EMG RMS value was significantly lower compared to group B 2 months postoperatively (*p* = 0.042), but group B’s orbicularis oculi RMS value was significantly lower compared to group A 12 months postoperatively (*p* = 0.020). Yet, no differences were found between groups for the normalized EMG values (RMS/maximal amplitude) and median frequency.

The median sEMG RMS and the median frequency of the frontal muscles and orbicularis oculi muscles are shown in Table 5. Group A’s 12 months post-upper-blepharoplasty RMS values had decreased significantly compared to baseline (*p* = 0.026). There were no significant differences in the normalized EMG outcomes (index RMS/maximal amplitude) during maximal contraction in the postoperative course compared to baseline.

During the maximal contraction period, the median frequencies had shifted at the end, becoming lower than at the start (Table 6), which indicates muscle fatigue. The median frequency shift seemed to improve postoperatively with time for group B’s frontalis and orbicularis oculi muscles. Group A only showed a decrease in median frequency shift in the frontalis muscle 12 months postoperatively. However, these pre- and postoperative differences in median frequency shifts were not significant.

### 3.4. Correlation

There was no significant correlation between the pre- and postoperative changes in the different variables (Table 7).

Regarding the baseline values and their correlation with changes in all variables, a significantly low positive correlation was found between the baseline eyebrow height and change in the 6 months postoperative HIT-6 values (r_s_(48) = 0.367, *p* = 0.009), but not in the 12-month follow-up values (r_s_(33) = 0.088, *p* = 0.617). There were no other significant correlations between the variables (see Table 7).

## 4. Discussion

In this study, we demonstrate a decrease in eyebrow height and in headache complaints after an upper blepharoplasty, regardless of whether only skin or skin with an additional strip of orbicularis oculi muscle is resected.

After an upper blepharoplasty, the frontalis muscles do not need to lift the eyebrows anymore to compensate for excessive eyelid skin. Subsequently, the frontalis muscles can relax and, as a result, the eyebrows tend to move down postoperatively. An anatomical and physiological relationship between the eyebrows, eyelid opening, and frontalis activation is suggested. When raising the eyebrows, the eyelid opening increases [24] which may be beneficial when the upper visual field is restricted or in the presence of heavy eyelids due to redundant upper eyelid skin.

Multiple studies have assessed the occurrence of brow ptosis after an upper blepharoplasty and, in general, the eyebrows tend to move down postoperatively [5], although not all studies have found significant differences between the pre- and postoperative measurements. However, the studies applied different methods to measure eyebrow height such as angular measurements [25], eyebrow height change reported as percentages [26] and ratios [27], or digitally calibrated measurements [15,28,29,30,31]. In addition, different landmarks such as the vertical eyebrow height at the exocanthion, endocanthion, mid-pupillary line, or lateral limbus were used. We chose the exocanthus as an anatomical landmark since it is a clear landmark that does not change after surgery.

Whether lowering the eyebrows has a negative effect on the aesthetic results is unclear. When the eyebrows move down postoperatively, the tarsal platform show may be less visible with time and may lead to a recurrence of excess upper eyelid skin. Whether this results in a softening of the forehead wrinkles is not clear. Another important factor is the shape and inclination of the eyebrows, since this affects eyebrow aesthetics [32]. It might be possible that the shape of the eyebrow is more important than the eyebrow height, and that patients are not really bothered by the lowering of the eyebrows postoperatively. The effect of lowering the eyebrow on the aesthetic results as perceived by patients has to be elucidated further in future studies.

In theory, preoperatively, continuous eyebrow elevation during the day may lead to problems such as tension-type headache. We found a low positive correlation [33] of 0.4 between the baseline eyebrow height and the postoperative change (after 6 months) in HIT-6 scores. This means that the higher the preoperative eyebrow, the more the HIT-6 score might be reduced.

Although the relationship between muscle activation and tension-type headache is controversial [3], we did find a significant improvement in headache complaints (HIT-6) 12 months postoperatively in both groups. This finding is in line with similar studies [20,34]. Castien et al. [21] proposed that a clinically relevant improvement in headaches is reflected by a decrease of at least 8 points on the HIT-6 questionnaire. Although both groups showed significant improvement in HIT-6 score, only group A showed a decrease of more than 8 points 12 months postoperatively, while group B only decreased by 4 points. However, in group B, the preoperative HIT-6 value was lower, so that a decrease of more than 8 points was not feasible.

We also found a significant decrease in group A’s RMS sEMG during maximal contraction 12 months after the upper blepharoplasty. This is an indication that the frontalis muscle requires less motor recruitment to elevate the eyebrow to the same height during maximal contraction compared to baseline. This is in line with the expectation of less local muscle fatigue (electrical activity) of the frontalis muscle postoperatively, but we do not know why we did not observe this in the skin/muscle group. One explanation could be that the delicate balance between the frontalis muscle and its antagonist orbicularis oculi muscle differs between the skin-only blepharoplasty and when the orbicularis oculi muscle is resected.

In general, during isometric contraction, muscle fatigue is accompanied by a decrease in motor unit firing rate. The EMG power density shifts to lower frequencies and, consequently, the median frequency decreases. As the muscle fatigues, additional fibres have to be recruited in order to generate the same force. This results in an increase in EMG amplitude and an increase in RMS values [18]. We assessed muscle fatigue using median frequencies. Muscle fatigue is generally defined as an activity-induced loss of the ability to produce force with the muscle and is often the result of prolonged use [35]. We hypothesized that, when the eyebrows are constantly raised preoperatively, the frontalis muscles might be at risk of muscle fatigue. However, the changes within the groups in median frequency were not significant during the course of this study. We also studied the median frequency shift in more detail by comparing the start of the maximal contraction with the end of the maximal contraction. Although we observed that the frequency shifts became smaller after surgery, which indicates less muscle fatigue, these differences are not significant. We therefore cannot prove that muscle fatigue changes substantially after a blepharoplasty.

Our study did not assess the levator palpebrae superioris muscle, whose primary function is to elevate the upper eyelid. Excess eyelid skin might lead to muscle fatigue and so the frontalis muscle is recruited to elevate the eyelid–eyebrow unit as a whole. However, we could not acquire an sEMG of this muscle due to practical difficulties. The surface EMG electrodes would interfere with normal eyelid opening and we would have had to resort to invasive techniques such as needle or wire electrode EMGs. In addition, due to the position of the muscle, it is difficult to acquire an EMG measurement. Kim et al. [1], who also concluded that upper blepharoplasty is associated with a gradual decrease in frontalis muscle activity, used needle electromyography. The disadvantage of needle-EMG is that only a small part of the muscle is recorded, whereas surface EMG covers a larger part of the muscle [36] and may therefore be more representative of the electrical activity of the muscle. The downside of surface EMG is that it suffers from crosstalk with neighbouring muscle activity, which can interweave with that of the target muscle [37]. This seems unlikely for the frontalis muscle. On the other hand, even the smallest electrode can potentially interfere with the movements of small muscles such as those of the face. The Kim et al. [1] study also used comparable methods to our study, such as normalized EMG data. The reason why we added the normalized (RMS/maximal amplitude) EMG values to our study was to compare the results better within and between the groups. The anthropomorphic differences between recording sites and between individuals might affect comparisons. These differences may include subcutaneous adipose tissue thickness, muscle resting length, contraction velocity, subtle changes in posture, interelectrode distance and impedance of the skin. However, the normalised EMG values did not differ between groups and within groups.

The non-normalised frontalis muscle RMS values showed that the electrical activity of the frontalis muscle was significantly lower in group A compared to group B two months postoperatively. This indicates that the skin-only participants’ muscles required less motor recruitment to elevate the eyebrow to the same height during maximal contraction (raising the eyebrows maximally) compared to the skin/muscle group. Although group A demonstrated lower electrical activity of the frontalis muscle, this did not lead to differences in patient-reported headaches between the groups.

Regarding the 12-month follow-up of orbicularis oculi muscle RMS results, the group B value was lower compared to group A. Therefore, it seems that the skin/muscle group needed less motor recruitment to achieve the same amount of muscle contraction compared to the skin-only group. This implies that a skin-only blepharoplasty possibly induces minor difficulties in contraction of the orbicularis oculi. However, it is important to mention that the differences between the groups regarding sEMG do not seem to be clinically relevant. When the regression coefficients were subjected to back-transformation with the log transformed raw values, an adjusted difference of −1.7 mV (RMS) in the of the frontalis muscle between groups was found, and 1.9 mV (RMS) in the orbicularis oculi muscle between groups. These differences are smaller than the intra-individual day-t-day variability (13% during maximal contraction) in healthy subjects [38], and therefore we consider them, although statistically significant, not clinically relevant.

A limitation of our study is that our patients only showed mild headache symptoms preoperatively, so we could not assess the effect of an upper blepharoplasty on moderate to severe headaches. In addition, the HIT-6 questionnaire was designed to provide a global measure and does not differentiate between various types and causes of headache. Another thought for future studies entails the eyebrow height measurements. Although our eyebrow measurements showed excellent repeatability, some improvements in eyebrow height measurements are possible. For example, the upper limit of the eyebrows could be used as a cut-off point, since this area is usually not subjected to eyebrow epilation. Future studies should standardise and make eyebrow height measurements uniform since a variety of methods have been used so far.

## 5. Conclusions

The eyebrow height decreased and patients reported less headaches after upper blepharoplasty irrespective of the used technique.

## Figures and Tables

**Table 1:** Patient characteristics after randomisation.

	Treatment A *n* = 27		Treatment B *n* = 27		*p* Value	
**Gender (number and % female)**	21 (78%)		23 (85%)		*0.484*	
**Age (years; mean ± SD [range])**	58 ± 8.6 [43–70]		55 ± 9.1 [39–70]		*0.241*	
**Dermatochalasis severity score (number of patients)**	*Right eye* Normal: 0 Mild: 11 Moderate: 15 Severe: 1	*Left eye* Normal: 0 Mild: 10 Moderate: 16 Severe: 1	*Right eye* Normal: 0 Mild: 12 Moderate: 13 Severe: 2	*Left eye* Normal: 0 Mild: 13 Moderate: 12 Severe: 2	*Right eye* *p = 0.771*	*Left eye* *p = 0.523*
**Removed skin (g; mean ± SD [range])**	*Right eye*	*Left eye*	*Right eye*	*Left eye*	*Right eye*	*Left eye*
	0.30 ± 0.08 [0.18–0.42]	0.32 ± 0.08 [0.21–0.51]	0.32 ± 0.11 [0.18–0.61]	0.34 ± 0.12 [0.14–0.65]	*p = 0.563*	*p = 0.703*
**Removed muscle (g; mean ± SD [range])**	*-*	-	*Right eye*	*Left eye*	-	
			0.11 ± 0.07 [0.05–0.40]	0.11 ± 0.07 [0.05–0.40]		
**Medial fat removal (no. of patients)**	2 *		0		*p = 0.552*	

* Removal of medial fat from both eyes.

**Table 2:** Median HIT-6 scores [Q1;Q3] and differences between groups.

	Group A Median [Q1;Q3] (*p*-Value *)	Group B Median [Q1;Q3] (*p*-Value *)	Adjusted Difference between Groups A and B ** (95% CI) and *p*-Value	
**Preoperatively**	46 [40;55]	42 [40;58]	*n.a.*	
**6 months postoperatively**	40 [36;44]	38 [36;45]	−2 (−7–3)	*p* = 0.383
	(*p* = 0.126)	(*p* = 0.052)		
**12 months postoperatively**	37 [36;42]	38 [36;41]	3 (−3–9)	*p* = 0.301
	(*p* = 0.003)	(*p* = 0.029)		

* *p*-value of the comparison between the preoperative and postoperative outcomes within a group (including Bonferroni correction). ** The adjusted difference is the regression coefficient from the generalised estimating equation models, which represents the difference in HIT-6 score between the treatment groups (group A–group B), after adjusting for baseline HIT-6 score, gender, age, dermatochalasis severity score, and amount of tissue removed.

**Table 3:** Eyebrow height in millimetres (median[Q1;Q3]): differences between groups.

	Preoperatively		6 Months Postoperatively	12 Months Postoperatively
	Group A Median [Q1;Q3]	Group b Median [q1;q3]	Adjusted Difference * between Groups a and b (95% ci) and *p*-Value	Adjusted Difference * between Groups A and B (95% CI) and *p*-Value
**Landmark a and a’**	15.8 [13.6;19.3]	16.5 [14.6;19.1]	−0.3 [−1.0;0.5] *p* = 0.502	0.1 [−0.9–1.1] *p* = 0.897
**Landmark b and b’**	16.7 [13.5;19.8]	17.5 [15.4;20.5]	−0.8 [−1.6;0.1] *p* = 0.082	−0.3 [−1.3;0.7} *p* = 0.575
**Landmark c and c’**	16.7 [13.2;18.6]	16.8 [15.2;19.7]	−0.7 [−1.8;0.3] *p* = 0.169	−0.4 [−1.6;0.7] *p* = 0.474

* The adjusted difference is the regression coefficient from the generalised estimating equation models, which represents the difference in eyebrow height (in millimetres) between the treatment groups (group A–group B), after adjusting for baseline eyebrow height, gender, age, dermatochalasis severity score, and amount of tissue removed.

**Table 4:** Eyebrow height in millimetres (median[Q1;Q3]): pre-and postoperative differences.

	Preoperatively	6 Months Postoperatively		12 Months Postoperatively	
	Median [Q1;Q3]	Median [Q1;Q3]	Difference Compared to Baseline (*p*-Value)	Median [Q1;Q3]	Difference Compared to Baseline (*p*-Value)
**Group A**					
**Landmark a and a’**	15.8	13.2	−2.6	13.6	−2.2
	[13.6;19.3]	[11.7;16.2]	(*p* < 0.001)	[11.4;16.9]	(*p* < 0.001)
**Landmark b and b’**	16.7	14.0	−2.7	13.1	−3.6
	[13.5;19.8]	[11.7;16.6]	(*p* < 0.001)	[11.7;16.7]	(*p* < 0.001)
**Landmark c and c’**	16.7	13.7	−3.0	12.4	−4.3
	[13.2;18.6]	[11.4;16.2]	(*p* < 0.001)	[11.1;16.4]	(*p* < 0.001)
**Group B**					
**Landmark a and a’**	16.5	14.4	−2.1	15.1	−1.4
	[14.6;19.1]	[13.0;16.4]	(*p* < 0.001)	[13.1;17.3]	(*p* < 0.001)
**Landmark b and b’**	17.5	14.6	−2.9	15.5	−2.0
	[15.4;20.5]	[12.8;16.7]	(*p* < 0.001)	[12.9;18.1]	(*p* < 0.001)
**Landmark c and c’**	16.8	14.3	−2.5	14.5	−2.3
	[15.2;19.7]	[11.9;16.8]	(*p* < 0.001)	[12.7;16.9]	(*p* < 0.001)

**Table 5:** Pre-and post-operative EMG values (median[Q1;Q3]) and differences between groups.

	Preoperatively	2 Months Postoperatively		6 Months Postoperatively		12 Months Postoperatively		
	Median [Q1;Q3]	MEDIAN [Q1;Q3] (*p*-Value *)	Adjusted Difference between Groups A and B ** (95% CI) and *p*-Value	Median [Q1;Q3] (*p*-Value *)	Adjusted Difference between Groups A and B ** (95% CI) and *p*-Value	Median [Q1;Q3] (*p*-Value *)	Adjusted Difference between Groups A and B ** (95% CI) and *p*-Value	Group
***RMS during maximal contraction (mV)***								
**Frontalis muscle**	80 [22;125]	73 [42;157] *p* = 0.255	−0.225 (−0.441–−0.008) *p* = 0.042	49 [29;97] *p* = 0.525	−0.131 (−0.338–0.076) *p* = 0.215	39 [18;107] *p* = 0.026	0.090 (−0.134–0.314) *p* = 0.430	Group A
**Frontalis muscle**	46 [32;73]	62 [23;113] *p* = 0.253		76 [43;123] *p* = 0.253		59 [27;119] *p* = 0.253		Group B
**Orbicularis oculi muscle**	51 [27;96]	44 [31;100] *p* = 0.145	−0.020 (−0.196–0.157) *p* = 0.826	51 [30;104] *p* = 0.145	−0.117 (−0.301–0.067) *p* = 0.211	40 [22;70] *p* = 0.145	0.282 (0.045–0.520) *p* = 0.020	Group A
**Orbicularis oculi muscle**	61 [22;100]	50 [28;96] *p* = 0.801		66 [34;118] *p* = 0.801		56 [36;111] *p* = 0.801		Group B
***Index (RMS/maximal amplitude) during maximal contraction (normalised value)***								
**Frontalis muscle**	0.21 [0.13;0.30]	0.21 [0.16;0.25] *p* = 0.392	−0.02 (−0.07–0.03) *p* = 0.386	0.18 [0.15;0.21] *p* = 0.392	−0.04 (−0.09–0.02) *p* = 0.225	0.19 [0.15;0.22] *p* = 0.392	−0.03 (−0.09–0.04) *p* = 0.459	Group A
**Frontalis muscle**	0.20 [0.12;0.27]	0.19 [0.14;0.22] *p* = 0.840		0.19 [0.13;0.22] *p* = 0.840		0.19 [0.16;0.24] *p* = 0.840		Group B
**Orbicularis oculi muscle**	0.21 [0.16;0.24]	0.20 [0.18;0.26] *p* = 0.187	−0.001 (−0.04–0.04) *p* = 0.958	0.17 [0.12;0.20] *p* = 0.187	0.03 (−0.01–0.07) *p* = 0.102	0.19 [0.16;0.22] *p* = 0.187	0.01 (−0.02–0.04) *p* = 0.666	Group A
**Orbicularis oculi muscle**	0.18 [0.16;0.21]	0.19 [0.17;0.23] *p* = 0.535		0.18 [0.13;0.22] *p* = 1.000		0.18 [0.14;0.22] *p* = 1.000		Group B
***Median frequency during maximal contraction (Hz)***								
**Frontalis muscle**	71 [62;85]	77 [60;100] *p* = 0.102	−0.066 (−0.134–0.001) *p* = 0.054	65 [59;74] *p* = 0.102	−0.037 (−0.090–0.016) *p* = 0.169	65 [59;77] *p* = 0.102	0.004 (−0.050–0.058) *p* = 0.889	Group A
**Frontalis muscle**	64 [56;72]	64 [60;74] *p* = 0.172		64 [57;69] *p* = 0.172		66 [58;100] *p* = 0.172		Group B
**Orbicularis oculi** **muscle**	100 [95;122]	110 [100;123] *p* = 0.724	−0.035 (−0.106–0.036) *p* = 0.337	106 [95;122] *p* = 0.724	−0.033 (−0.085–0.020) *p* = 0.222	108 [100;118] *p* = 0.724	0.044 (−0.009–0.096) *p* = 0.101	Group A
**Orbicularis oculi muscle**	98 [73;116]	100 [80;114] *p* = 0.086		97 [71;106] *p* = 0.086		110 [100;124] *p* = 0.086		Group B

* *p*-value of the comparison between preoperative and postoperative outcomes within a group (including Bonferroni correction). ** The adjusted difference is the regression coefficient from the generalised estimating equation models, which represents the difference in HIT-6 score between the treatment groups (group A–group B), after adjusting for baseline values, gender, age, dermatochalasis severity score, and amount of tissue removed. The raw values were transformed before undertaking the generalised estimating equations analysis. The estimates of the adjusted differences represent differences in the transformed scale (i.e., 10 log) for the ‘RMS during maximal contraction’ and ‘Median frequency during maximal contraction’ values. The ‘Index (RMS/maximal amplitude)’ values were not transformed.

**Table 6:** Median frequency shift during maximal contraction (Fmed shift).

**Group A**	**Frontalis Muscle**				**Orbicularis Oculi Muscle**			
	**Median Frequency (Hz)**	**Median Frequency (Hz)**	**Difference (%)**	*p*-Value *	**Median Frequency (Hz)**	**Median Frequency (Hz)**	**Difference (%)**	*p*-Value *
	**Start**	**End**			**Start**	**End**		
**Preoperatively**	71	67	−5.6 %	-	112	101	−9.8%	-
	[62;79]	[58;77]			[91;133]	[70;121]		
**2 months postoperatively**	75	69	−8.0%	0.381	124	105	−15.3%	0.345
	[61;126]	[58;102]			[103;146]	[80;133]		
**6 months postoperatively**	69	66	−4.3%	0.381	106	99	−6.6%	0.345
	[60;75]	[57;77]			[84;125]	[64;121]		
**12 months postoperatively**	66	69	+4.5%	0.381	111	97	−12.6%	0.345
	[57;83]	[59;85]			[96;130]	[52;117]		
**Group B**	**Frontalis muscle**				**Orbicularis oculi muscle**			
	**Median frequency (Hz)**	**Median frequency (Hz)**	**Difference (%)**	*p*-value *	**Median frequency (Hz)**	**Median frequency (Hz)**	**Difference (%)**	*p*-value *
	**Start**	**End**			**Start**	**End**		
**Preoperatively**	65	56	−13.8%	-	95	67	−29.5%	-
	[54;71]	[51;71]			[67;113]	[55;98]		
**2 months postoperatively**	65	58	−10.8%	0.331	110	85	−22.7%	0.417
	[57;76]	[53;73]			[90;125]	[54;106]		
**6 months postoperatively**	62	60	−3.2%	0.331	97	91	−6.2%	0.417
	[55;73]	[53;71]			[63;107]	[58;106]		
**12 months postoperatively**	63	65	+4.8%	0.331	114	105	−7.9%	0.417
	[57;85]	[57;96]			[98;135]	[77;128]		

* *p*-value of the comparison between the postoperative median frequency shift and the baseline (preoperative) median frequency shift.

**Table 7:** Correlation between variables.

	Change in Headache (r_s_ and *p*-Value)	Change in Eyebrow Height (r_s_ and *p*-Value)	Change in EMG Frontalis Muscle (r_s_ and *p*-Value)
**Correlation between the changes (pre- and postoperative values) in the different variables**			
** *6-month follow-up* **			
**Change in headache**	-	−0.060, *p* = 0.681	−0.083, *p* = 0.615
**Change in eyebrow height**	−0.060, *p* = 0.681	-	0.080, *p* = 0.627
**Change in EMG frontalis muscle**	−0.083, *p* = 0.615	0.080, *p* = 0.627	-
			
**Correlation between baseline values and pre- and postoperative changes in the variables**			
**Baseline headache**	-	−0.123, *p* = 0.394	−0.078, *p* = 0.653
**Baseline eyebrow height**	0.367, *p* = 0.009	-	0.133, *p* = 0.420
**Baseline EMG frontalis muscle**	0.292, *p* = 0.071	−0.134, *p* = 0.414	-
**Correlation between the changes (pre- and postoperative values) in the different variables**			
** *12-month follow-up* **			
**Change in headache**	-	−0.043, *p* = 0.814	−0.115, *p* = 0.630
**Change in eyebrow height**	−0.043, *p* = 0.814	-	0.136, *p* = 0.465
**Change in EMG frontalis muscle**	−0.115, *p* = 0.630	0.136, *p* = 0.465	-
			
**Correlation between baseline values and pre- and postoperative changes in the variables**			
**Baseline headache**	-	0.097, *p* = 0.518	0.193, *p* = 0.291
**Baseline eyebrow height**	0.088, *p* = 0.617	-	0.056, *p* = 0.760
**Baseline EMG frontalis muscle**	0.154, *p* = 0.473	−0.195, *p* = 0.261	-

## Data Availability

The data presented in this study are available on request from the corresponding author.
